# Probing binding and occlusion of substrate in the human creatine transporter‐1 by computation and mutagenesis

**DOI:** 10.1002/pro.4842

**Published:** 2024-01-01

**Authors:** Amy Clarke, Clemens V. Farr, Ali El‐Kasaby, Daniel Szöllősi, Michael Freissmuth, Sonja Sucic, Thomas Stockner

**Affiliations:** ^1^ Institute of Pharmacology and the Gaston H. Glock Research Laboratories for Exploratory Drug Development, Center of Physiology and Pharmacology Medical University of Vienna Vienna Austria; ^2^ Department of Theoretical and Computational Biophysics Max Planck Institute for Multidisciplinary Sciences Göttingen Germany

**Keywords:** creatine transporter, cysteine 144, molecular dynamics simulations, protonation

## Abstract

In chordates, energy buffering is achieved in part through phosphocreatine, which requires cellular uptake of creatine by the membrane‐embedded creatine transporter (CRT1/SLC6A8). Mutations in human *slc6a8* lead to creatine transporter deficiency syndrome, for which there is only limited treatment. Here, we used a combined homology modeling, molecular dynamics, and experimental approach to generate a structural model of CRT1. Our observations support the following conclusions: contrary to previous proposals, C144, a key residue in the substrate binding site, is not present in a charged state. Similarly, the side chain D458 must be present in a protonated form to maintain the structural integrity of CRT1. Finally, we identified that the interaction chain Y148‐creatine‐Na^+^ is essential to the process of occlusion, which occurs via a “hold‐and‐pull” mechanism. The model should be useful to study the impact of disease‐associated point mutations on the folding of CRT1 and identify approaches which correct folding‐deficient mutants.

## 1 INTRODUCTION

Adenosine triphosphate (ATP) is the universal cellular energy currency (Fiske & Subbarow, 1929a; Lipmann, 1941; Lohmann, 1929): all energy consuming reactions exert a drain on cellular ATP levels. Moreover, cellular ATP has been posited to play a role in proteostasis by acting as a hydrotrope and preventing protein aggregation, for which millimolar concentrations of ATP are required (Greiner & Glonek, 2021; Mandl et al., 1952; Patel et al., 2017). Accordingly, cells have adopted phosphagens to buffer cellular ATP‐levels during periods of excess ATP demand (Sauer & Schlattner, 2004). Phosphocreatine, the first phosphagen to be discovered (Eggleton & Eggleton, 1927a, 1927b; Fiske & Subbarow, 1929b), is used by chordate cells. Phosphocreatine is produced by the Lohmann reaction, in which creatine kinase catalyzes the transfer of the γ‐phosphate from ATP to creatine, generating adenosine diphosphate (ADP) and phosphocreatine in the process (Lohmann, 1934).

The hydrolysis of phosphocreatine results in a larger free energy change (−45.0 kJ mol^−1^) than the conversion of ATP to ADP (−31.8 kJ mol^−1^) (Wyss & Kaddurah‐Daouk, 2000). As a result, cells such as neurons and striated muscle, which undergo spikes of high energy demand, must accumulate large concentrations of creatine to support the formation of adequate levels of phosphocreatine (up to 32 mM; Kushmerick et al., 1992) and to maintain an effective ATP‐buffering system. Most cells in the human body cannot synthesize creatine: their creatine supply originates from dietary intake and from synthesis in hepatocytes (Wyss & Kaddurah‐Daouk, 2000) and in a limited number of cells in the brain (Braissant & Henry, 2008). Thus, most cells require uptake of creatine from the extracellular fluid, where creatine is present at concentrations ranging from 30 to 100 μM. In brain and striated muscle, creatine accumulates to intracellular levels of about 5 mM (Rackayova et al., 2017) and 7 mM (Kushmerick et al., 1992), respectively. This uphill transport is accomplished by CRT1, a member of the solute carrier 6 family (SLC6) (Guimbal & Kilimann, 1993). CRT1 utilizes the Na^+^/Cl^−^ concentration gradient to actively transport one creatine molecule per one Cl^−^ and two Na^+^ ions (Farr et al., 2022; Peral et al., 2002). Like all of the membrane transporters, the creatine transporter (SLC6A8) cycles through a series of conformational states, including an extracellularly facing outward‐open (OO) state, a sealed occluded state, and an inwardly facing inward‐open state, in order to translocate creatine across the lipid bilayer (Drew & Boudker, 2016; Jardetzky, 1966).

Pathogenic, loss‐of‐function mutations in CRT1 lead to creatine transporter deficiency (CTD). CTD is associated with intellectual disability, autism, epilepsy, and delayed speech acquisition (Farr et al., 2020). Thus far, more than 80 loss‐of‐function mutations in CRT1 that lead to CTD have been identified (Farr et al., 2020; Salazar et al., 2020). These mutations can exert their mechanistic effects through varied ways: of the individual point mutations which have been investigated, the vast majority causes protein misfolding (El‐Kasaby et al., 2019; Salazar et al., 2020). However in some instances, the mutations allow for surface delivery of the protein but appear to interfere with substrate binding, resulting in reduced transport activity (Salazar et al., 2020). The current treatment strategy for CTD is based on supplementation with creatine, but this approach shows only limited benefits. However, recent research indicates that, in some instances, the folding defect can be corrected by chemical chaperoning (El‐Kasaby et al., 2019).

An experimentally solved structure of CRT1 would provide a valuable basis for understanding how CRT1 function is impaired by disease‐causing mutations. As this structure is currently not available, homology modeling can serve as a powerful, computational alternative. For CRT1, the solution of multiple structures of closely related SLC6 transporters, such as the human serotonin transporter (SERT) (Coleman et al., 2016, 2019), provides a strong foundation for homology modeling. Moreover, the generated homology models can then be used as a starting point for subsequent molecular dynamics simulations. These simulations can help refine and validate the predicted structure, by informing on side chain orientation and the ionization states of titratable residues, as well as providing greater information on the dynamics of protein motion. This information is not only absent from computationally derived homology models, but also often from empirically solved structures.

Here, we exploit the computational triad of homology modeling, molecular dynamics, and docking to generate a stable homology model of the human creatine transporter using SERT as a template. We validated the model using molecular dynamics simulations and probed the ionization states of key titratable residues through experiment and computation. From this, we show that D458 is present in an uncharged state in CRT1, and contrary to previous proposals (Colas, 2020; Colas et al., 2020; Colas & Laine, 2021; Dodd & Christie, 2001; Stary & Bajda, 2023), C144 is not present in a charged state in the substrate binding site. Using this homology model as a starting point, we then docked creatine, the cognate substrate of CRT1, into the binding site. We show that residues F68, G73, and Y148 are important for mediating the interaction of CRT1 with creatine and for also triggering the process of transporter occlusion, an essential first step in the transport process.

## 2 RESULTS

### 2.1 SERT can be used to generate a homology model of CRT1

**FIGURE 1:**
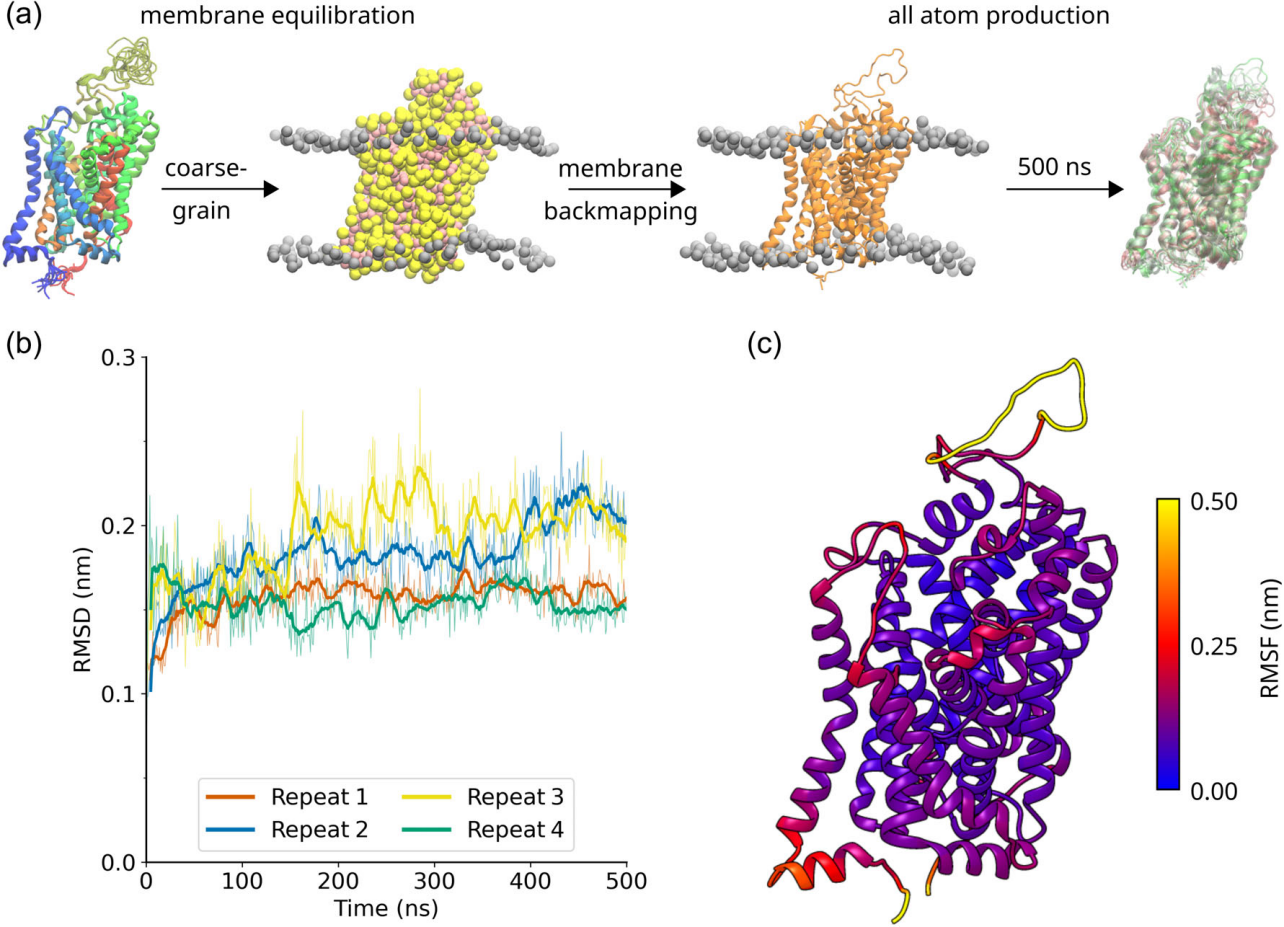
Workflow of the molecular dynamics simulations. (a) Four homology models of CRT1 were generated with the MODELLER program using the outward‐open serotonin transporter structure (PDB: 5I73) as a template. The models were converted to a coarse‐grained representation and subjected to a 1 μs simulation. The resulting models were then converted back to an all‐atom representation and simulated for 500 ns using the amber99sb‐ildn force field. Refinement of the protonation states of ionizable residues and analysis of the homology models occurred during this stage. (b) Root mean square deviation (RMSD) calculations for the Cα atoms of the transmembrane helices (helices 1–12) for all the atom simulations, after least squares fitting on the scaffold domain. The bold lines indicate the running average, with a window of 10 ns applied. The thin lines indicate the raw data. (c) Root mean square fluctuation (RMSF) calculation projected onto the tertiary structure of the transporter. The RMSF is an average of four repeats.

In order to probe the structural dynamics of CRT1, including its interaction with creatine, we devised a computational workflow involving homology modeling, molecular dynamics simulations, and docking (Figure 1a). To begin, we generated homology models using the human SERT (protein data bank (PDB): 5I73) in an OO conformation as a template. SERT was chosen as a template for homology modeling due to the high sequence identity between CRT1 and SERT (44%), and due to the availability of multiple experimentally solved SERT structures. In the course of sequence alignment, we identified a unique aspartate (D458) on the second turn of transmembrane (TM) helix 9 (TM9). In other members of the SLC6 family, this aspartate is usually a serine or glycine residue (Figure S1). Given its position in a hydrophobic, membrane‐embedded turn of the helix, we chose to protonate D458 so that it was present in an uncharged form, as previous molecular dynamics simulations have shown that the membrane environment can modulate the pKa of aspartate residues (Panahi & Brooks, 2015).

One hundred homology models of CRT1, including the ion cofactors, were generated using the MODELLER program (Webb & Sali, 2016). The four homology models with the lowest molpdf score were then selected for 500 ns simulations according to the scheme in Figure 1a. Two important metrics for assessing the stability of a homology model in a molecular dynamics simulation are root mean square deviation (RMSD) and root mean square fluctuation (RMSF) calculations. RMSD calculations illustrate the extent to which the homology model deviates from the starting structure during the course of the simulation. Calculating the RMSD for the Cɑ atoms of TMs 1–12 showed that the deviation from the starting structure was generally small (<0.25 nm) and reached a plateau after around 200 ns (Figure 1b). In contrast, RMSF captures the fluctuations of atoms around their average position. RMSF calculations showed that the homology models display behavior typical of transmembrane proteins: the Cɑ atoms of the transmembrane helices underwent little fluctuation (<0.25 nm, Figure 1c), compared to the loops and termini, whose fluctuations were more pronounced (>0.25 nm, Figure 1c).

### 2.2 D458 is present in a neutral state in the transporter

**FIGURE 2:**
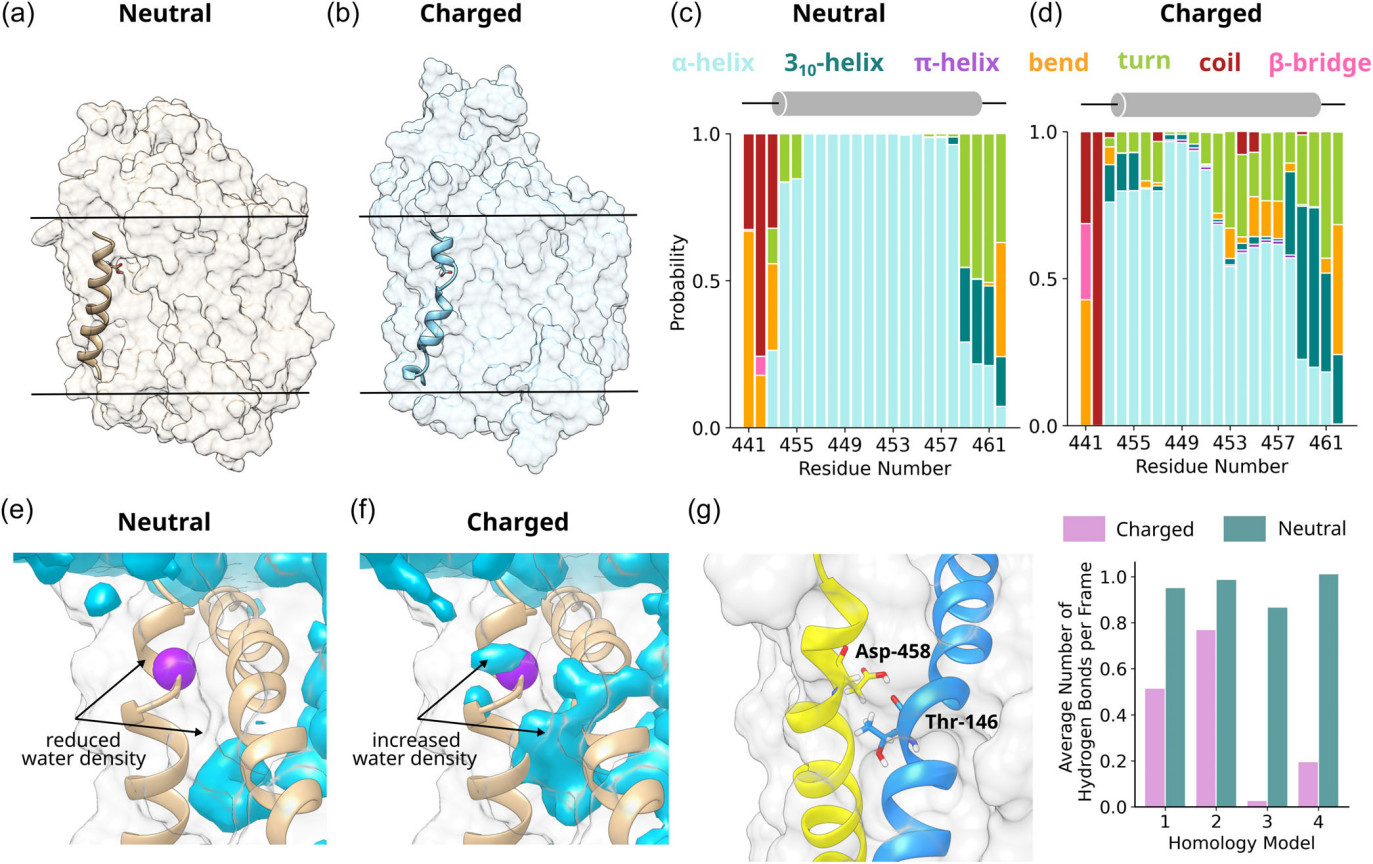
D458 protonation stabilizes transmembrane helix 9 (TM9). (a, b) Image showing the structure of TM9 in the presence of a neutral D458 (a) and a charged D458 (b). The hydrophobic boundary of the bilayer is indicated with a black line. (c, d) Bar graph showing the probability of residues 441–462 being in different secondary structural states for the neutral (c) and charged (d) D458, as defined by the Dictionary of Protein Secondary Structure (DSSP) algorithm (Kabsch & Sander, 1983). The data are averaged over four repeats. (e, f) Water density analysis showing water density in the presence of a (e) neutral aspartate and a (f) charged aspartate. In both cases, the data were averaged over four repeats. The location of D458 is indicated with a purple sphere. (h) A protonated aspartate side chain forms a hydrogen bond with the backbone carbonyl of T146, as shown in the left panel. The right panel shows the average number of hydrogen bonds per frame between D458 and T146 for the charged (pink) and neutral (teal) protonation states.

We confirmed that D458 was present in a protonated state by repeating the simulations with D458 in the charged state. Aspartate residues rarely populate TM helices due to the instability of a negative charge in the highly hydrophobic membrane environment (Chamberlain et al., 2003; Ulmschneider & Sansom, 2001). The impact of aspartate residues on membrane‐inserted α‐helices has been examined by both experimental (Caputo & London, 2004) and computational approaches (Panahi & Brooks, 2015). These document that: (i) the membrane environment has a substantial effect on the pKa of the side chain (Panahi & Brooks, 2015) and (ii) that depending on its position within the helix, the charged state of aspartate can preclude membrane insertion (Caputo & London, 2004). Indeed, in our simulations, the negative charge of D458 led to significant structural breakdown compared to the neutral D458, with the ɑ‐helix of TM9 decomposing into turn‐ and bend‐like structures during the course of the simulation (Figure 2a–d). For example, when D458 was neutral, residue 453 was in an ɑ‐helix form for 100% of the simulation time, compared to only 54% when D458 was charged. This is likely due to the charged carboxylate attracting significant water to the inter‐helix space between TM3 and TM8 (Figure 2e,f), which disrupts the inter‐helix interaction network. In contrast, in the neutral form, the carboxylic acid moiety of D458 is able to form a hydrogen bond with the backbone carbonyl oxygen of T146 of TM3 (Figure 2g). Not only does neutralization reduce water influx into the inter‐helix space, but the neutralized side chain of D458 acts as an interaction point between TM3 and TM8, suggesting that the protonation of D458 is important for maintaining the structural integrity of the scaffold domain.

**FIGURE 3:**
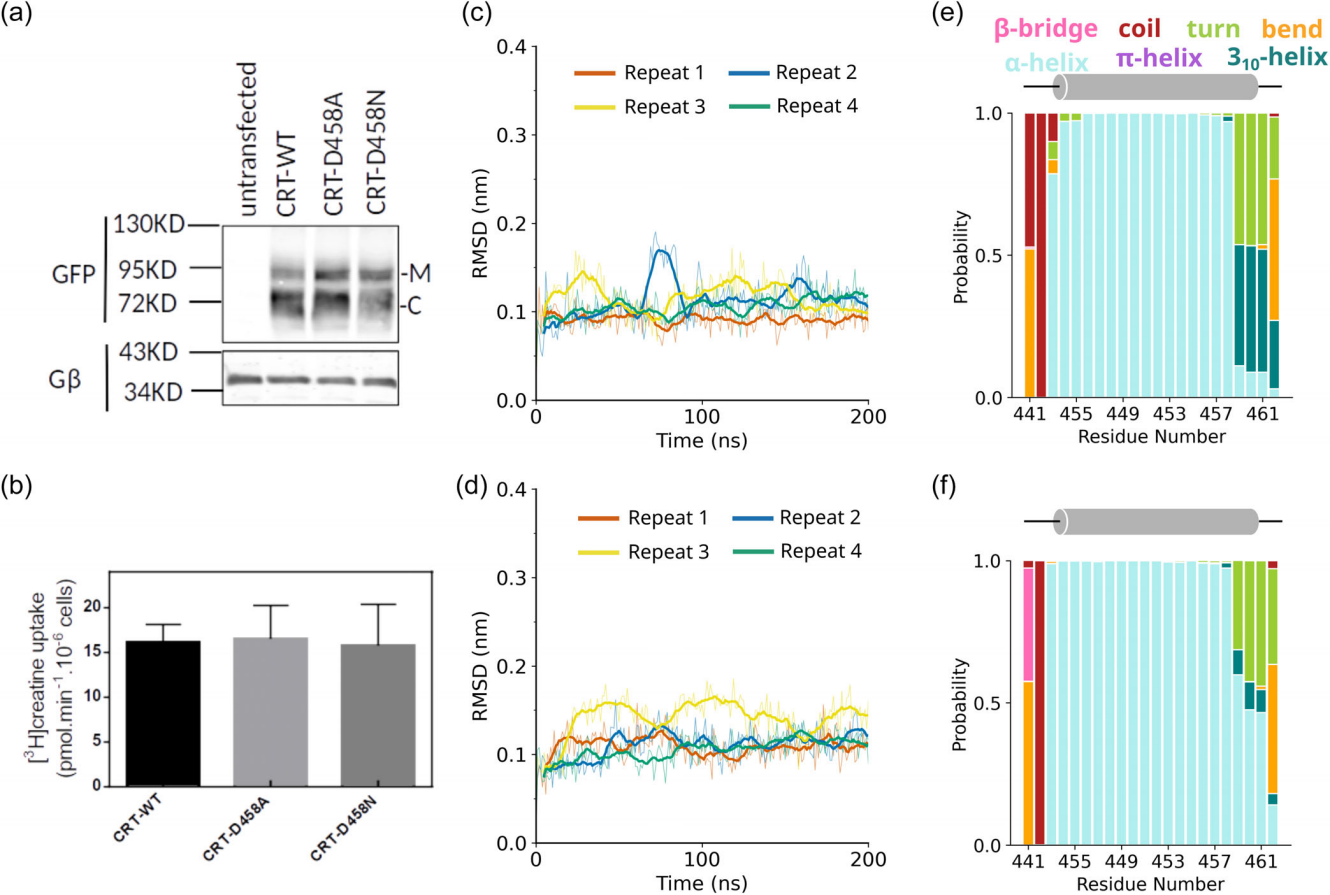
Experimental and computational evidence to support the neutral protonation state of D458. (a) Immunoblotting for wild‐type CRT1 (WT), CRT1‐D458A (D458A), and CRT1‐D458N (D458N). Aliquots (10 μg) of lysates, which had been prepared from untransfected HEK293 cells and HEK293 cells transiently expressing YFP‐tagged wild type and mutant transporters, were resolved by denaturing electrophoresis. After transfer to nitrocellulose membranes, the immunoreactive bands were detected with an antibody directed against green fluorescent protein (GFP) (upper blot) or the G protein β‐subunits (as a loading control, lower blot). M and C indicate the positions of the (ER‐resident) core‐glycosylated species and the transporters harboring mature glycan moieties, respectively. (b) Substrate uptake velocity of wild‐type CRT1 (WT), CRT1‐D458A (D458A), and CRT1‐D458N (D458N). HEK293 cells were transfected with plasmids encoding YFP‐tagged wild type and mutant versions of CRT1. After 24 h, cells (2 × 10^5^/well) were seeded onto poly‐D‐lysine‐coated 48‐well plates and allowed to adhere overnight. Subsequently, the uptake reaction was initiated by adding 3 μM [^3^H]creatine and carried out for 6 min as described in Section 5. Non‐specific uptake was determined in the presence of 300 μM β‐guanidinopropionic acid (<5% of total uptake) and was subtracted. Data are means ± standard deviation (SD) from the three independent experiments carried out in triplicate. (c, d) Root mean square deviation (RMSD) analysis showing the stability of TM9 in the (c) CRT1‐D458A and (d) CRT1‐D458N. The bold lines indicate the running average, with a window of 10 ns applied. (e, f) Bar graph showing the probability of residues 441–462 being in different secondary structural states for (e) CRT1‐D458A and (f) D458N, as defined by the DSSP algorithm (Kabsch & Sander, 1983). The data were averaged over four repeats.

We verified the prediction that the neutral state of D458 was important by creating two experimental mutants, CRT1‐D458A and ‐D458N. If the charge of D458 is required for the structural integrity of and/or substrate translocation by CRT1, mutation to alanine or asparagine should result in reduced expression of the mutated transporter and reduced transport velocity. First, we assessed the expression levels of wild‐type CRT1 and of the mutant variants after transient expression in HEK293 cells. CRT1 harbors several putative N‐ and O‐linked glycosylation sites, resulting in heterogeneity of electrophoretically migrating species. Band assignment to the species carrying mature glycanes and the core glycane (labeled M and C, respectively, in Figure 3a) is based on deglycosylation experiments (El‐Kasaby et al., 2019). It is evident from Figure 3a that the mature (i.e., transport‐competent) form of CRT1‐D458A and CRT1‐D458N accumulated to levels comparable to that of wild‐type CRT1. Likewise, after transient expression in HEK293 cells, substrate uptake velocity was comparable for all three transporter variants (Figure 3b). Moreover, 200 ns simulations of the D458A and D458N mutants were consistent with the experimental data: secondary structure analysis calculations showed that these mutants were equivalent to the wild‐type transporter (cf. Figures 2c,d and 3e,f), and RMSD calculations showed TM9 is stable for both mutants (Figure 3c,d). Taken together, our combined molecular dynamics and experimental data confirm that the unique D458 residue is present in its neutral form. This is consistent with its presence in a hydrophobic, bilayer‐embedded turn of TM9, where charged residues reside very infrequently (Chamberlain et al., 2003; Ulmschneider & Sansom, 2001).

### 2.3 C144 is present in a neutral state in the S1

**FIGURE 4:**
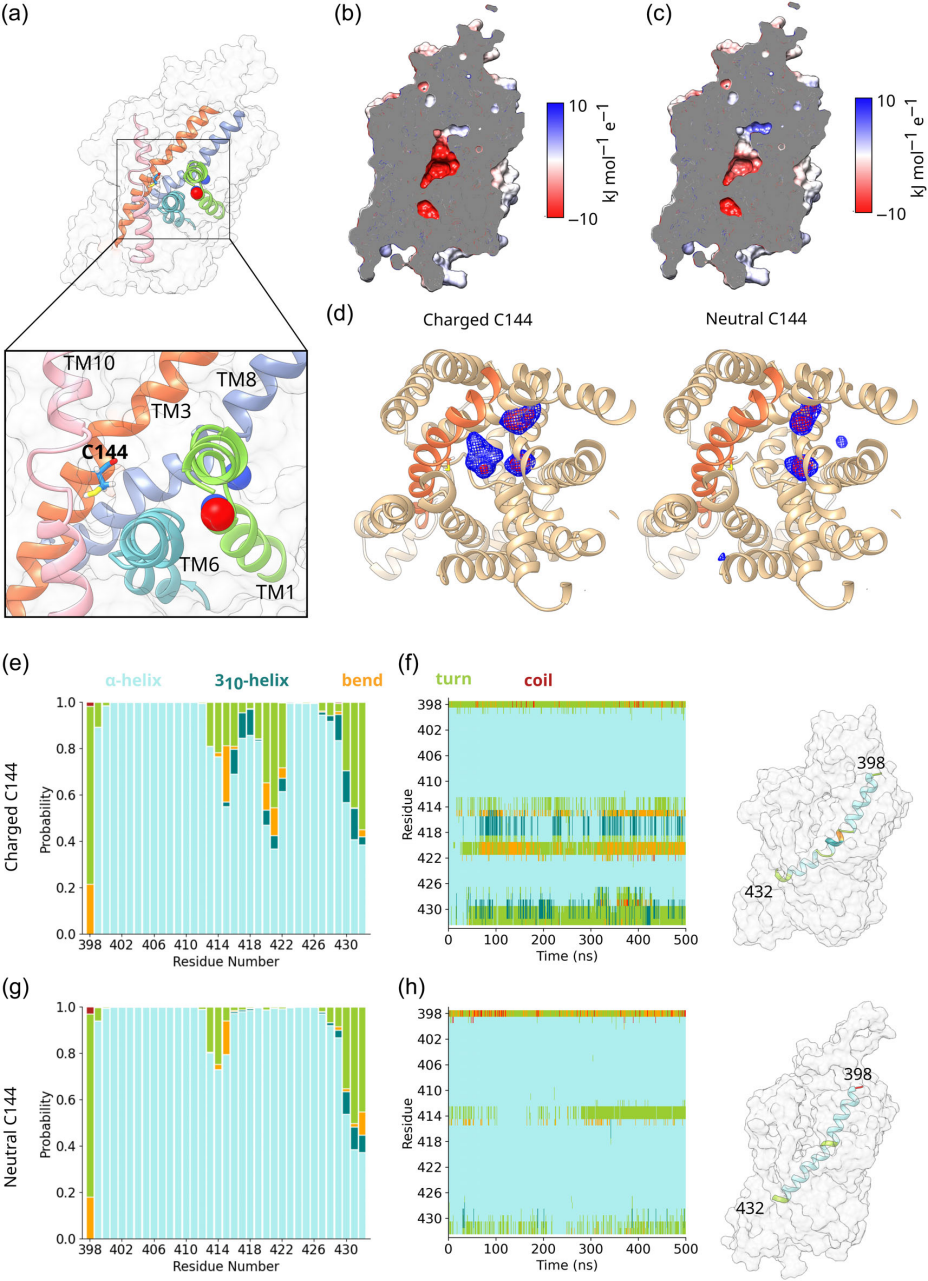
In its charged state, C144 causes local structural destabilization. (a) Image showing the location of C144 midway up TM3, projecting into the substrate binding site. For clarity, only those helices which line the substrate binding site (TM1, TM3, TM6, TM8, and TM10) are shown. (b, c) Cutaway of CRT1 with the results of an Advanced Poisson‐Boltzmann Solver calculation projected onto its surface, for the (b) charged and (c) neutral states of C144. (d) Volume map showing Na^+^ density for the charged (left panel) and neutral (right panel) states of C144. TM helices are shown in tan, with the exception of TM3, which is shown in orange. The data were averaged over four repeats. (e, g) Bar graph showing the probability of residues 399–432 of TM8 being different secondary structural elements for the (e) charged and (g) neutral states of C144. The data were averaged over four repeats. (f, h) Time‐resolved secondary structure plot, showing the time evolution of the secondary structure of TM8 for the (e) charged and (g) neutral states of C144, as defined by the DSSP algorithm (Kabsch & Sander, 1983). A representative example is shown in each case, whereby the TM8 segments are colored according to secondary structure. For visualization, the cardinal ribspline option of UCSF chimera was used.

Another ionizable residue that is of interest to the function of CRT1 is C144, which is found in TM3 in a position facing the substrate binding site (S1) (Figure 4a). The role of C144 in creatine transport was first probed in cysteine‐scanning experiments (Dodd & Christie, 2001), which showed that reaction of C144 with 2‐aminoethyl methanethiosulfonate (MTSEA) resulted in inactivation of the transporter. Due to the rapid reaction of C144 with MTSEA, it was proposed that C144 was present in the deprotonated, thiolate form (Dodd & Christie, 2001). In fact, the deprotonated state of C144 has repeatedly been referred to as a distinguishing feature of the CRT1 transport mechanism (Colas, 2020; Colas et al., 2020; Colas & Laine, 2021; Stary & Bajda, 2023), although since the original publication there has not been any further experimental or computational exploration of the protonation state of C144.

Given the location of C144 in a turn on TM3 facing the substrate binding site, we probed its protonation state before conducting docking experiments. PROPKA calculations (Olsson et al., 2011) predict the pKa of C144 to be approximately 12, higher than the typical pKa value of 9 for a cysteine residue, suggesting C144 is protonated. To confirm this, we ran four independent 500 ns simulations with C144 in a deprotonated state and compared them to the four simulations of CRT1 with a protonated C144. Advanced Poisson‐Boltzmann Solver (APBS; Baker et al., 2001) calculations showed that the presence of a deprotonated Cys and therefore negatively charged cysteine created a strongly electronegative substrate binding site (Figure 4b,c). The result of this strong electronegative potential was increased Na^+^ density in the vicinity of C144 (Figure 4d). This density resulted from both the unbinding of Na^+^ from the Na1 binding site, and from the entry of a third Na^+^ from the extracellular medium into the substrate binding site. The effect of additional Na^+^ density in the vicinity of TM3 was the local structural destabilization of TM8 (Figure 4e–h), which resulted in partial ɑ‐helix unwinding and the formation of additional bend and turn.

**FIGURE 5:**
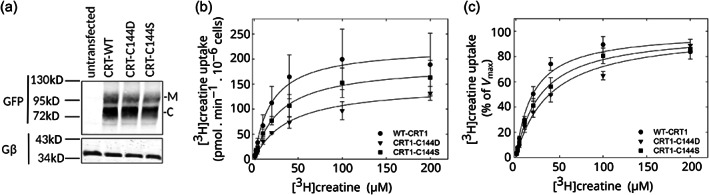
Experimental evidence against deprotonation of C144 in the substrate binding pocket of CRT1. (a) Immunoblotting for wild‐type CRT1 (WT), CRT1‐C144D (C144D), and CRT1‐C144S (C144S). Aliquots (10 μg) of lysates, which had been prepared from untransfected HEK293 cells and HEK293 cells transiently expressing YFP‐tagged wild type and mutant transporters, were processed as outlined in legend to Figure 2a. After transfer to nitrocellulose membranes, the immunoreactive bands were detected with an antibody directed against GFP (upper blot) or the G protein β‐subunits (as a loading control, lower blot). M and C indicate the positions of the (ER‐resident) core‐glycosylated species and the transporters harboring mature glycan moieties, respectively. (b) Saturation curves for substrate by wild‐type CRT1 (WT‐CRT1, circles), CRT1‐C144D (triangles), and CRT1‐C144S (squares). HEK293 cells were transfected with plasmids encoding YFP‐tagged wild type and mutant versions of CRT1. After 24 h, cells (2 × 10^5^ /well) were seeded onto poly‐D‐lysine‐coated 48‐well plates and allowed to adhere overnight. Subsequently, the uptake reaction was initiated by adding [^3^H]creatine at the indicated concentration and carried out for 6 min as described under Section 5. Non‐specific uptake was determined in the presence of 300 μM β‐guanidinopropionic acid (<5% of total uptake) and was subtracted. Data are means ±SD from the three independent experiments carried out in triplicate. (c) Replot of the data shown in panel (b): uptake was normalized to the *V*_max_ (=100%) calculated from fitting a hyperbola to the data points obtained in each individual uptake experiment. This normalization allowed for accounting for inter‐experimental variation in transfection efficiency and thus allowed for illustrating the rightward shift in *K*_m_ resulting from the C144D mutation, which introduced a charge into the substrate binding pocket.

The computational data suggested that the presence of a charged C144 caused local structural instability. Hence, we examined the impact on transporter levels and function of substituting C144 with serine and with aspartate. When transiently expressed in HEK293 cells, the mature glycosylated form of the mutants CRT1‐C144S and CRT1‐C144D accumulated to levels modestly lower than that of the wild‐type CRT1 (Figure 5a). Similarly, the maximum substrate uptake velocity was on average reduced by about 15% and 33% for CRT1‐C144S and CRT1‐C144D, respectively, when compared to *V*_max_ of the wild‐type transporter (Figure 5b; Table S1). More importantly however, the mutation C144D altered substrate affinity. This is most readily evident in Figure 5c, where the individual curves were normalized to their *V*_max_‐values extracted from the curve fitting: the *K*_m_ for creatine of CRT1‐C144D (39.1 ± 6.4 μM, mean ± standard deviation (SD), *n* = 3) was higher than the *K*_m_ of wild‐type CRT1 (19.4 ± 2.4 μM) (Table S1). This difference was statistically significant (*p* = 0.016, analysis of variance (ANOVA) followed by post hoc Holm‐Šídák pairwise multiple comparison). In contrast, the *K*_m_ of CRT1‐C144S (29.3 ± 6.9 μM) did not differ from that of wild‐type CRT1 (*p* = 0.146) and the difference between CRT1‐C144S and CRT1‐C144D failed to reach the threshold of statistical significance (*p* = 0.077).

It is important to note that the *K*_m_ for substrate uptake is a composite parameter, affected by many rate constants. Kinetic modeling shows that it differs substantially from the true substrate affinity, that is, the affinity of the substrate for the outward open state (Schicker et al., 2022). Due to the absence of high affinity inhibitors of CRT1, it is not possible to carry out the radioligand binding experiments which would allow for an estimation of the substrate affinity to the outward open state of CRT1‐C144S and CRT1‐C144D. However, the increase in *K*_m_ seen with CRT1‐C144D, albeit modest, indicates that substrate affinity is reduced by introducing a residue which is expected to be charged in the water‐filled substrate binding site. In contrast, the side chain of serine is not considered to be ionizable within the physiologically relevant pH range. Thus, taken together, these observations suggest that C144 is present in an uncharged, neutral form in the binding site of CRT1.

### 2.4 Docking and subsequent molecular dynamics simulations can be used to identify the binding mode of creatine

**FIGURE 6:**
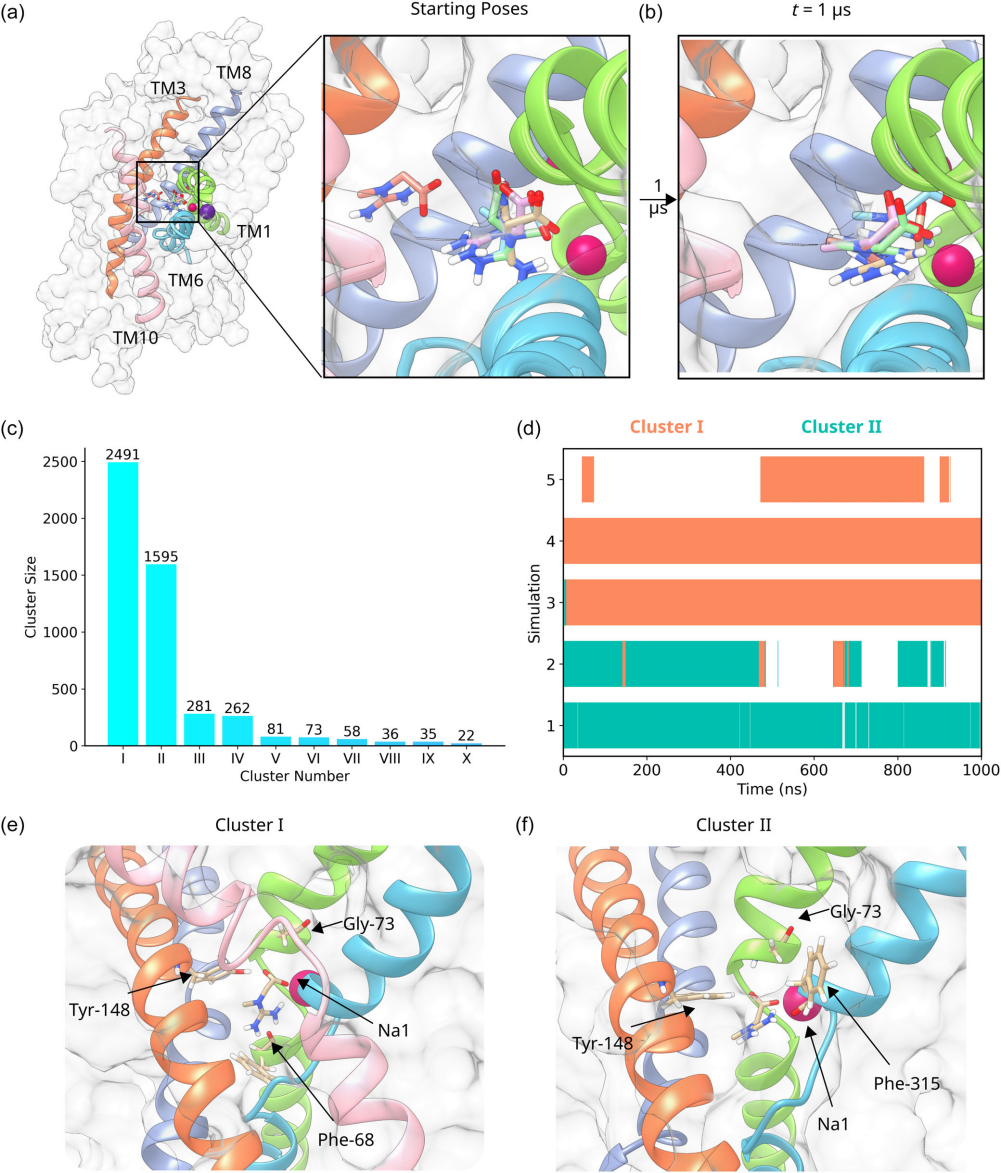
Docking of creatine to CRT1. (a) The five docked poses generated by the docking program HADDOCK. Creatine is shown in a stick representation. CRT1 is shown in a surface representation and only the TM helices which line the substrate binding site are shown in a ribbon representation. (b) The five poses at the end of a 1 μs unbiased simulation. (c) Bar graph showing the size of the 10 largest clusters as identified through the single‐linkage clustering algorithm implemented in GROMACS. For clarity, only the 10 largest clusters are shown. (d) Event plot indicating the time points at which a creatine pose corresponds to either Cluster I (orange) or Cluster II (teal) for the production run of the simulations. White indicates membership of another cluster. (e) The pose corresponding to Cluster I. The four interacting residues are shown in a stick representation. The color of the helices corresponds to the coloring in (a). (f) The pose corresponding to Cluster II. The four interacting residues are shown in a stick representation. The color of the helices corresponds to the coloring in (a).

After generating a stable homology model and probing the ionization states of key ionizable residues, we used the High Ambiguity Driven protein‐protein DOCKing (HADDOCK) docking (Honorato et al., 2021; van Zundert et al., 2016) to investigate the interaction between creatine and CRT1. Figure 6a illustrates the top poses of the five best clusters, as determined by the HADDOCK scoring function: it is evident that the HADDOCK program generated a diverse set of poses (Figure 6a). In order to scrutinize these poses, we used them as the starting point for a 1 μs all‐atom simulation. This timescale is long enough for the bound creatine to reorient itself to a more favorable binding pose, if the initial binding pose is incorrect. In addition, previous molecular dynamics simulations showed that this timescale is suitable for capturing the transition toward the occluded state for SLC6 transporters (Gradisch et al., 2022). Observing such a convergence is an important step in verifying the binding poses: a transition to the occluded state would only occur if creatine engaged in the correct interactions with CRT1.

Interestingly, in all five cases the final pose at the end of the 1 μs simulation is different from the starting pose (Figure 6b). In several cases, the creatine molecule reoriented during the equilibration procedure. This suggests that the docking did not identify the optimal interactions between creatine and CRT1. In order to explore which creatine–CRT1 interactions were dominant in the simulations, all five simulations were clustered using the single‐linkage clustering algorithm implemented in GROMACS (Figure 6c). Thirty‐six clusters were identified in total: the largest cluster (Cluster I) contained 2491 structures, which were nearly 50% of the total number of structures. The second largest cluster (Cluster II) contained 1595 structures. As a result, nearly 82% of the structures were therefore contained within Clusters I and II, indicating the simulations converged on two dominant creatine‐binding poses during the production run (Figure 6d).

The Cluster I structure corresponds to a binding pose where creatine interacts with residues from both the scaffold (TM helices 3, 4, 8, and 9) and bundle domains (TM helices 1, 2, 6, and 7). In the Cluster I pose, creatine interacts with the scaffold domain via a hydrogen bond with the hydroxyl group of Y148 from TM3. It interacts with the bundle domain via hydrogen bonds with F68 and G73 of TM1 (see Figure 6e). Creatine also forms an electrostatic interaction with Na1, located between TM1 and TM6. This binding pose is consistent with the binding modes of other transporters of the SLC6 family: it has been shown that ligand interaction with elements of both the scaffold and bundle domain is a requirement for triggering the process of occlusion (Gradisch et al., 2022). The Cluster II pose differs from the Cluster I pose in that the guanidinium group does not interact with F68 from TM1 but rather with F315 from TM6 (Figure 6f).

Although Cluster I represented the largest cluster containing nearly 50% of the total structures, this pose was not identified as a top pose by the HADDOCK docking program. As a comparison, AutoDock 4 (Morris et al., 2009) was also used to dock creatine into the S1 (Figure S2). The five best docked poses generated by AutoDock 4, according to the AutoDock scoring function, were less diverse than the HADDOCK docked poses. In all five cases, the guanidinium group interacted with residues from TM1 and TM6, and there was no interaction between the carboxyl group and either Y148 or the Na1 ion. However, as with HADDOCK, none of these poses correspond to those of Cluster I, which we identified by molecular dynamics simulations. It is possible that this reflects weaknesses in the respective docking scoring functions, or the lack of full backbone and sidechain flexibility in docking runs, which are important for ligand binding, regardless if it occurs by induced fit or by conformational selection.

The molecular dynamics simulations summarized in Figure 6 identified several interactions between CRT1 and creatine. The acid test to verify their importance is to examine whether they can trigger the process of transporter occlusion. The mechanism of occlusion of the closely related SERT has been previously extensively studied by Gradisch et al. (2022). In this model of substrate‐triggered occlusion, serotonin interacts with SERT through a “hold‐and‐pull” mechanism. Interactions at their energetic minima “hold” the indole moiety of the serotonin molecule in a position between TM3 and TM8 of the scaffold domain, while the ethylamine moiety of serotonin exerts a “pull” on the bundle domain of SERT by interacting with residues from TM1 and TM6. The “pulling” or attractive forces between the serotonin molecule and the bundle domain drive the movement of the bundle domain toward the scaffold domain. The resulting reduction in inter‐helix distance allows for closure of the outer hydrophobic gate. The hydrophobicity of serotonin further facilitates the movement of TM1 and TM6 by causing water efflux from the S1, generating space for the helices to move into.

**FIGURE 7:**
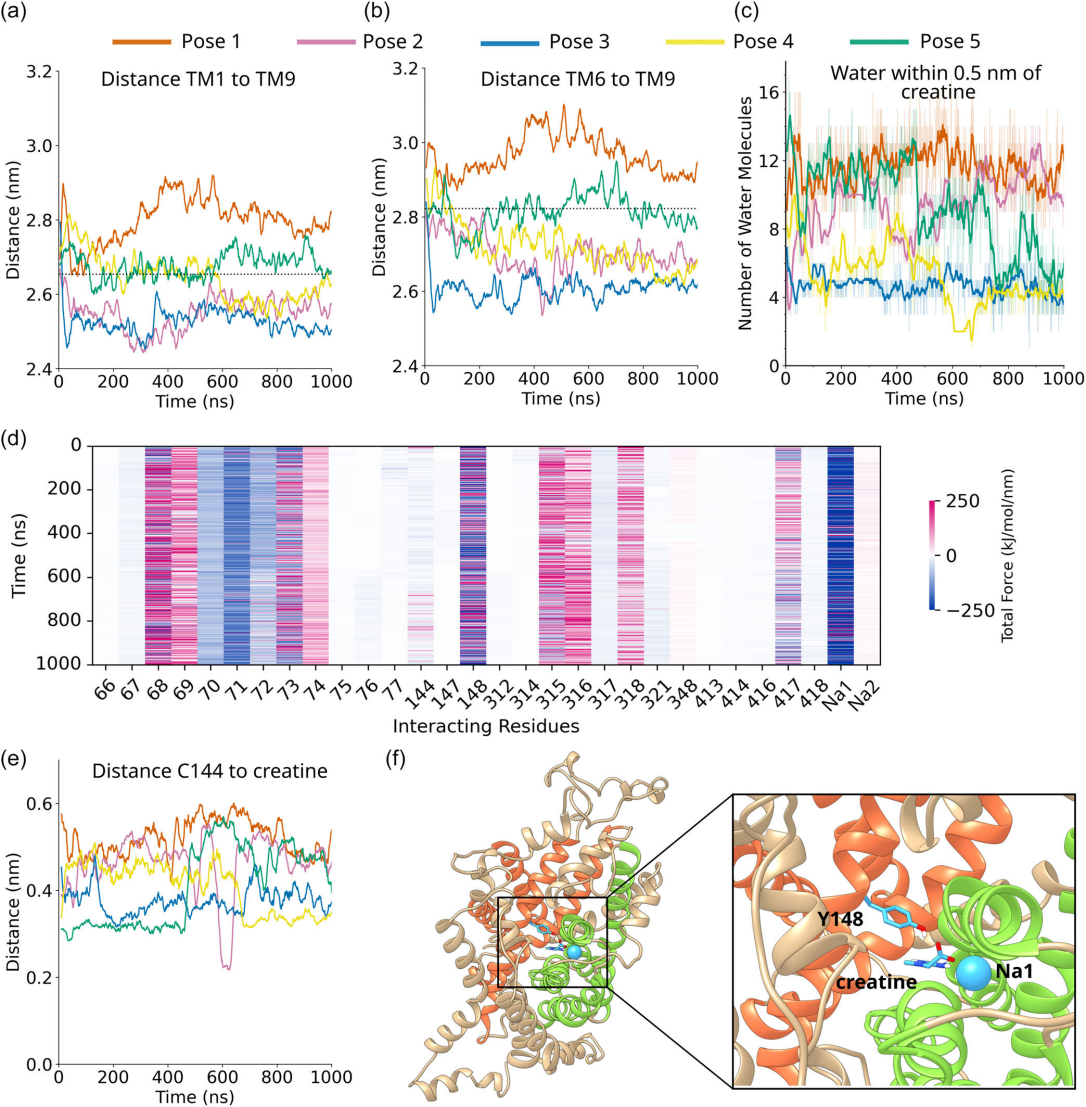
Simulation 4 undergoes a transition toward the occluded state. (a) Distance measurement between the center of mass of residues 72–85 of TM1b and 456–458 of transmembrane helix 9 (TM9). A window average of 10 ns was applied. The dotted line indicates the distance between these helices in the occluded conformation of serotonin transporter (SERT). (b) Distance measurement between the center of mass of residues 303–317 of TM6a and 456–458 of TM9. A window average of 10 ns was applied. The dotted line indicates the distance between these helices in the occluded conformation of SERT. (c) Number of water molecules within 0.5 nm of creatine as a function of time. The bold lines indicate the running average, with a window of 10 ns applied. The thin lines indicate the raw data. (d) Time‐resolved force distribution analysis for simulation 4, the occluding trajectory. The 30 residues which exert the greatest force on creatine are shown and colored according to the total force (electrostatic plus van der Waals) exerted on creatine. (e) Smallest distance between creatine and C144 as a function of time for the five simulations. A window average of 20 ns has been applied. (f) Interaction chain of Y148—creatine—and Na^+^ in the first sodium ion binding site (Na1). Helices are colored according to whether they are part of the scaffold domain (orange), bundle domain (green), or neither (tan).

From this model of SERT occlusion, three important observables for monitoring the course of transporter occlusion can be derived: firstly, a reduction in the distance between TM1 and TM9; secondly, a reduction in the distance between TM6 and TM9; thirdly, a decrease in the amount of water in the substrate binding site. Applying these three metrics to the five creatine‐bound CRT1 simulations indicated that the simulation corresponding to starting pose 4 showed both the characteristic reductions in inter‐helix distances and a reduction of water in S1 (Figure 7a–c). The distance between TM1b and TM9 decreased to <2.65 nm, which in the homologous SERT is an indicator of the occluded transporter state (Coleman et al., 2019; Gradisch et al., 2022). Similarly, the distance between TM6a and TM9 decreased to <2.82 nm. This suggests that simulation pose 4 has reached an occluded state. Although simulation pose 3 also showed the characteristic reduction in inter‐helix distance and water count in the S1, the conformations of the trajectory indicated anomalous secondary structure changes, including partial unwinding of TM6b. These are not associated with the typical pathway of occlusion. As a result, we did not include simulation 3 in our further analysis of the process of occlusion.

The combined analysis of interhelical distance and water count suggested that the interaction of creatine with CRT1 led to the occlusion in the simulation starting from pose 4. The creatine pose in simulation 4 corresponds to the Cluster I bound pose (Figure 5d), in which creatine makes contact with residues F68, G73, and Y148 of CRT1 and also the Na^+^ at binding site 1. This suggests that Cluster I not only represents the largest identified cluster, but also a binding mode that can result in transporter occlusion. We propose that this binding mode therefore represents a biologically relevant set of interactions between creatine and CRT1, although it is also possible that other binding modes could also lead to transporter occlusion on longer timescales.

According to the “hold‐and‐pull” mechanism described in Gradisch et al. (2022), interactions between the ligand and both the scaffold and bundle domains are essential for triggering occlusion. We explored the conjecture that this mechanistic principle also held true in CRT1 by using force distribution analysis (FDA) (Costescu & Gräter, 2013) to calculate the forces between CRT1 and creatine for the occluding trajectory (Figure 7d). FDA revealed that the interaction between Y148 of the scaffold domain and creatine was at its energetic minimum, with the force oscillating between −250 and +250 kJ mol^−1^ nm^−1^. Thus, the interaction had reached a (transient) equilibrium, which translated into a constant distance between the center of mass of Y148 and creatine. This is consistent with this residue “holding” the carboxyl group of creatine in its place. In contrast, the Na^+^ in the Na1 binding site exerts a strong “pulling” or attractive force on creatine, with forces in the region of −200 to −250 kJ mol^−1^ nm^−1^. This Na^+^ cofactor, an essential co‐transported ion, is coordinated by residues from TM1 and TM6 of the bundle domain. It is possible that this Na^+^ ion mediates the pulling effect in the “hold‐and‐pull” mechanism of occlusion.

Interestingly, the FDA analysis also showed that—with a force between 0 and −40 kJ mol^−1^ nm^−1^—C144 only exerted a modest total force on the creatine molecule in comparison to Y148 or the Na^+^ ion. This suggests that C144 does not play a critical role in transporter occlusion, a finding in line with the uptake experiments, which show that this position can accommodate mutation to serine without large changes in *K*_m_ (cf Figure 5). In order to confirm this conjecture, we calculated the smallest distance between C144 and creatine as a function of time (Figure 7e): the distances were greater than 3.5 Å, which indicates that C144 is not involved in hydrogen bonding with creatine, but rather interacts via hydrophobic interactions. Although hydrophobic interactions can still be important contributors to protein–ligand interactions, they are weak compared to electrostatic or hydrogen bonding interactions. Indeed, our simulations instead point to the interaction chain of Y148‐creatine‐Na1 as key determinants of creatine binding and transporter occlusion (Figure 7f).

## 3 DISCUSSION

The importance of CRT1 in humans is readily gauged from the phenotypic consequences of loss‐of‐function mutations, which cause intellectual disability, autism, epilepsy, and delayed speech acquisition in the affected (male) individuals (Farr et al., 2020). Progress in developing treatment strategies is hampered by the lack of pharmacological tools, such as high‐affinity ligands which could qualify as pharmacochaperones (Freissmuth & Stockner, 2018). These obstacles can be overcome by structure‐based docking campaigns, such as virtual screens (Boytsov et al., 2023), but this approach requires a structural model of CRT1. Here, we combined homology modeling with molecular dynamics simulations and experimental verification to develop a credible structural model of CRT1. To the best of our knowledge, our approach presents for the first time an extensive molecular dynamics exploration of the structure and dynamics of CRT1. Three major insights were gained: (i) the combination of docking and molecular dynamics simulations allowed for deducing the binding mode of creatine in the substrate binding pocket. (ii) The model illustrated a plausible route to substrate translocation by showing that substrate occlusion was accomplished by a “hold‐and‐pull” mechanism in a manner analogous to that seen in the related SERT. (iii) The model allowed for an exploration of the protonation state of two residues, C144 in TM3 and D458 in TM9. These residues are unique to CRT1 and are of interest to understand the role of ionizable sidechains in substrate binding and in the stability of the hydrophobic core of the transporter.

The predictions of our computational model are supported by previous experimental data: mutation of Y148 to a cysteine has been shown to result in an inactive transporter, although CRT1‐Y148C accumulated to expression levels comparable to that of wild‐type CRT1 (Dodd & Christie, 2005). Our model of the process of CRT1 occlusion suggests that the interaction chain of Y148‐creatine‐Na^+^ in the Na1 binding site is essential to the process of transporter occlusion, with Y148 “holding” creatine while creatine in turn pulls on Na1. A cysteine at the Y148 position would not be able to form a hydrogen bond with the carboxyl group of creatine, and so a critical link in the interaction chain would be lost, leading to transporter inactivation. This model is also consistent with experimental data that shows that CRT1 binds creatine and the co‐transported ions in a highly cooperative manner (Farr et al., 2022). Our computational data suggests this cooperativity could come, in part, from the essential interaction between creatine and Na^+^ in the Na1 binding site.

The correct protonation states of ionizable residues is essential for maintaining the proper structure and function of membrane proteins. pKa is sensitive to the local microenvironment, such as the presence of other charged or polar residues, water, or hydrophobic residues and molecules. For example, changes in the microenvironment of a critical aspartate residue of bacteriorhodopsin allow the pKa value of this residue to range from 2.6 to 9.7 (Balashov et al., 1996). Molecular dynamics has demonstrated that incorrect ionization states of titratable residues in membrane proteins can lead to artifactual effects, such as spurious Na^+^ binding in SERT (Chan et al., 2022). Previously, C144 was proposed to exist in a charged, deprotonated state in the substrate binding site; this was posited to be a key determinant of substrate binding (Colas, 2020; Colas et al., 2020; Colas & Laine, 2021; Dodd & Christie, 2001; Stary & Bajda, 2023). Our observations refute this hypothesis on three grounds. Firstly, computational analysis showed that the deprotonation of C144 resulted in local secondary structure degradation, due to an increased electronegativity in the substrate binding site. Secondly, our experimental and computational data also suggested that C144 did not play a critical role in the process of occlusion, with FDA analysis showing that C144 did not exert strong forces on creatine. Third, a negatively charged C144 would likely attract a third sodium ion to the S1, which could compete with the positively charged guanidinium moiety of creatine. This is predicted to reduce the affinity of substrate, which was, in fact, observed with CRT1‐C144D.

Similarly, our molecular dynamics simulations indicated that the hydrophobic environment around D458 precluded ionization of its side chain. Forcing deprotonation of D458 resulted in destabilization of the structure of TM9 and in the accumulation of water molecules. Conversely, replacing D458 by asparagine or alanine (non‐ionizable residues) neither impaired expression of the protein nor the velocity of substrate translocation. However, it is important to note that the experimental and computational tools available for studying the protonation states of ionizable residues are limited. The D458N and D458A mutations suggest that D458 is protonated, and the C144S and C144A suggest the same for C144, however these mutants are blunter changes than the addition of a proton. As a result, these data should be interpreted cautiously, although the convergence of both the computational and experimental data is supportive of a real effect.

Although in the future, our model could be used as a starting point to explain experimental models of CTD (Duran‐Trio et al., 2021; Fernandes‐Pires & Braissant, 2022; van de Kamp et al., 2013), for example by introducing point mutations and monitoring the resultant effect on protein structure and stability, certain challenges to understanding the structure–function effect of deleterious mutations of CRT1 using our homology model remain. Firstly, many of the pathogenic mutations arise from base deletions, which result in frameshifts. For example, D458 is the site of a pathogenic mutation: a four base deletion in exon 9 (coding sequence bases 1372–1375) was found in an affected patient (van de Kamp et al., 2013). This results in substitution serine for D458. However, because of the concomitant frameshift, it is not possible to draw any immediate conclusions from this mutation. Similarly, a three base deletion in exon 6 results in a deletion of F315, a residue shown in our study to be important for substrate binding, leading to a CTD characterized by extreme mental retardation (Fons et al., 2008; van de Kamp et al., 2013). Secondly, SLC6 folding in the endoplasmic reticulum occurs in the inward‐open conformation. As our homology model is in the OO conformation, understanding folding defects would require a new inward‐open model, although in this regard, the recent solution of the inward‐open and inward‐occluded structures of γ‐aminobutyric acid (GABA) transporter 1 (Motiwala et al., 2022; Nayak et al., 2023; Zhu et al., 2023) could provide good templates.

Our research highlights the importance of using molecular dynamics simulations to validate proposed homology models and docking poses. It is notable that neither docking program used in our computational analysis correctly identified the major binding mode predicted by molecular dynamics simulations. Moreover, while molecular dynamics simulations of CRT1 have not been performed before, two previous studies have used homology models of CRT1 as the starting point for docking (Colas et al., 2020; Salazar et al., 2020). In both cases, the docked pose with the highest score differs from the Cluster I bound pose identified in our molecular dynamics simulations, although the study from Colas et al. (2020) predicts a binding mode similar to our Cluster II pose. The difference between the docked poses and molecular dynamics pose could be due to several reasons, many of which have been previously extensively discussed (Kitchen et al., 2004; Sousa et al., 2006). With their focus on high‐throughput determination of binding poses, the scoring functions used in docking are inherently limited in comparison to the full physics‐based force fields of molecular dynamics simulations. In addition, unlike in molecular dynamics simulations, during docking the protein does not have full sidechain and backbone flexibility. It is possible that the homology model needs to undergo a period of equilibration before the correct interactions with creatine can be made. Finally, docking uses an implicit water model, as opposed to the explicit water model used in our molecular dynamics simulations. Given that the role of water has been shown to be extremely important in the process of occlusion for SLC6 transporters (Gradisch et al., 2022), this could be a particularly pertinent limitation of molecular docking in the case of CRT1.

## 4 CONCLUSIONS

The research we present here provides a starting point for further investigations on the process of substrate‐mediated transporter occlusion of the SLC6 family of transporters. Our data indicate that CRT1 uses the “hold‐and‐pull” mechanism as described for SERT occlusion. In this mechanism, interactions with the scaffold domain “hold” the ligand while attractive forces between the ligand and bundle domain pull the bundle domain toward the scaffold domain. Here, we propose that Y148 “holds” creatine while the pulling effect is mediated through the cofactor ion Na^+^ residing in the NA1 binding site. Thus, despite evolving separate cellular functions—the SERT is essential in neurotransmission, whereas CRT1 is an essential component of energy metabolism—the two transporters exploit the same underlying principles to achieve transporter occlusion.

The power of homology modeling has been extensively discussed since the release of AlphaFold2 (Jumper et al., 2021; Varadi et al., 2022). Against this backdrop of a growing interest in homology modeling, our data highlight the strengths of combining homology modeling with molecular dynamics simulations and experiments in order to test stability and confirm predictions. Given the clinical importance of CRT1, the model which we presented here provides an important tool in the quest for a repertoire of CRT1 ligands. These may ultimately translate into compounds suitable for pharmacotherapeutic approaches. Moreover, the emergence of further experimental structures of SLC6 transporters, such as the recent inward‐open and inward‐occluded structures of the GABA transporter 1 (Motiwala et al., 2022; Nayak et al., 2023; Zhu et al., 2023), will only aid in future model refinement.

## 5 MATERIALS AND METHODS

### 5.1 Materials

Cell culture media and supplements were purchased from Invitrogen. Bovine serum albumin and Complete™ protease inhibitor mixture were purchased from Roche Applied Science (Penzberg, Germany), polyethylenimine (PEI; linear 25 kDa) from Santa Cruz Biotechnology (Dallas, TX, USA), [^3^H]creatine (creatine [N‐methyl‐^3^H], specific activity 75 Ci mmol^−1^) from American Radiolabeled Chemicals (St. Louis, MO, USA), β‐guanidinopropionic acid (GPA) from Sigma–Aldrich (St. Louis, MO, USA), sodium dodecyl sulfate (SDS) from BioMol (Hamburg, Germany), scintillation fluid (Rotiszint® eco plus), and Tris from Carl Roth (Karlsruhe, Germany). The rabbit polyclonal anti‐green fluorescent protein (GFP) antibody (ab290) was obtained from Abcam (Cambridge, UK). A rabbit polyclonal antibody directed against a sequence common to all G protein β‐subunits (Hohenegger et al., 1996) was used for immunostaining to verify equivalent loading of gel lanes. The secondary antibody (Donkey anti‐rabbit, IRDye 680RD) was obtained from LI‐COR Biotechnology GmbH (Bad Homburg, Germany). All other chemicals used in experiments were of analytical grade.

### 5.2 Homology modeling

Homology models of CRT1 in the OO conformation were created using MODELER 9.24 (Webb & Sali, 2016) with the human SERT (PDB: 5I73) (Coleman et al., 2016) as a template. One‐hundred homology models were generated and the four models with the lowest molpdf score were selected for simulation.

### 5.3 Molecular dynamics simulations

Those homology models selected for simulation were truncated to residues 46–603 to remove the extended and flexible N‐ and C‐termini. In accordance with SERT, E490 was protonated (Chan et al., 2022). Homology models were then coarse‐grained using the martinize.py script (version 2.4; http://cgmartini.nl/images/tools/martinize/martinize-2.4/martinize.py) and embedded in a phosphatidylcholine/cholesterol (70/30) membrane with a box size of 10.7 × 10.7 × 10.4 nm, using the insane.py script (http://www.cgmartini.nl/images/tools/insane/insane.py) (Wassenaar et al., 2015). The system was solvated and NaCl was added to a final concentration of 150 mM. The systems were then simulated for 1 μs in order to generate an equilibrated lipid–protein system, using the Martini 2.2 force field for proteins and Martini 2.0 force field for lipids (de Jong et al., 2013; Marrink et al., 2004, 2007). The simulations were run with the standard Martini molecular dynamics parameters.

The equilibrated membrane was then converted to an all atom representation using backward.py (Wassenaar et al., 2014) and the homology model structure reinserted using the membed procedure (Wolf et al., 2010). All atom simulations were then run using the amber99sb‐ildn force field (Lindorff‐Larsen et al., 2010) with the GROMACS simulation package version 2019.2 (Abraham et al., 2015; Hess et al., 2008; Van Der Spoel et al., 2005). After 50 rounds of energy minimization using the steepest descent algorithm, four rounds of equilibration were performed for 0.5 ns each, with consecutively decreasing position restraints on the protein heavy atoms and co‐factor ions (1000, 100, 10, and 1 kJ). During the production run no position restraints are present on the protein or any other molecule. For the all atom simulations, a 2 fs timestep for integration was used, and coordinates were saved every 10 ps. The simulations were run at 310 K, maintained using the v‐rescale temperature coupling algorithm (Bussi et al., 2007). Pressure coupling at 1.0 bar in a semi‐isotropic manner was achieved using the Parrinello–Rahman barostat (Parrinello & Rahman, 1981). The Particle Mesh Ewald (PME) algorithm was used for electrostatic interactions with a cut‐off of 0.9 nm. A single cut‐off of 0.9 nm was used for van der Waals interactions. Creatine parameters were obtained by using existing atom types for aspartate and arginine from the amber99sb‐ildn force field. Partial charges were then obtained from the R.E.D. server (Bayly et al., 1993; Dupradeau et al., 2010; Vanquelef et al., 2011), using the default parameters.

Analysis of the resultant trajectories used scripts written in‐house. RMSD and RMSF calculations were performed with the GROMACS rms and rmsf modules, respectively. Secondary structure analysis used the GROMACS do_dssp module in conjunction with the Dictionary of Protein Secondary Structure (DSSP) program (Kabsch & Sander, 1983). The hydrogen bonding analysis and cluster analysis used the GROMACS hbond and cluster modules. In both cases, the standard options, such as cutoff distance, were used. Water density around TM9 and ion density in the substrate binding site were both calculated using the VolMap plugin of the visual molecular dynamics (VMD) program. APBS calculations used the APBS program (Baker et al., 2001). Distances were calculated using MDAnalysis (Gowers et al., 2016; Michaud‐Agrawal et al., 2011). Structural images were created using University of California, San Francisco (UCSF) chimera (Pettersen et al., 2004) and graphs were plotted using the matplotlib module of Python.

### 5.4 Molecular docking

Molecular docking was carried out using the HADDOCK web server version 2.4 (Honorato et al., 2021; van Zundert et al., 2016) (first accessed on December 30, 2022) and AutoDock 4 (Morris et al., 2009). For both sets of docking, the Cl^−^ and two Na^+^ cofactors were present, and the truncated protein (residues 46–603) was used. For the HADDOCK docking, the active residues used were F68, C144, A318, and G421 (Dodd & Christie, 2007). These residues have been confirmed experimentally to be important determinants of creatine specificity. All other parameters were kept as the default HADDOCK run parameters, with the exception of the “filter_buried” option which was set to FALSE.

### 5.5 DNA constructs and cloning

The cDNA encoding the wild‐type human CRT1 with a YFP moiety fused to the C‐terminus (El‐Kasaby et al., 2019) was used as template to create the CRT1‐D458A, CRT1‐D458N, CRT1‐C144D, and CRT1‐C144S with the QuikChange Lightning Site‐Directed Mutagenesis Kit (Stratagene, La Jolla, CA, USA). The presence of the mutation was confirmed by sequencing on both strands (LGC Labor GmbH Augsburg, Germany). It has previously been shown that C‐terminal tagging does not affect function of the channel and results in *K*_m_ and *V*_max_ values comparable to WT (El‐Kasaby et al., 2019). Similarly, a previous study has shown that *K*_m_ values derived from uptake experiments are comparable to those obtained by electrophysiology (Dai et al., 1999; Dodd et al., 2010; Schloss et al., 1994).

### 5.6 Cell culture and transfections

HEK293 cells were cultured in Dulbecco's modified Eagle's medium with high glucose (4.5 g L) and L‐glutamine (584 mg L^−1^), supplemented with 10% fetal calf serum. Transfections were done using PEI (1 mg mL^−1^): the plasmid DNA (10 μg) was combined with 30 μL PEI solution, allowed to incubate for 15 min at room temperature and then added dropwise to subconfluent cultures of HEK293 cells (70% confluency in a 10 cm dish).

### 5.7 Creatine uptake assays

Twenty‐four hours after transfection, HEK293 cells were detached, harvested, and seeded onto pre‐coated with poly‐D‐lysine 48‐well plates (2 × 10^5^/well). On the next day, the medium was removed, cells were washed twice with 1 mL Hanks' balanced salt solution (Sigma 55037C) and incubated for 30 min at 37°C in Hank's balanced salt solution. The cells were then washed twice with uptake buffer (10 mM HEPES·NaOH, pH 7.4, 4.7 mM KCl, 2.2 mM CaCl_2_, 1.2 mM MgSO_4_, 10 mM glucose, and 120 mM NaCl). For uptake experiments, the plate was placed in 37°C water bath, and cells were incubated in uptake buffer containing the indicated concentrations of [^3^H]creatine (specific activity adjusted with unlabeled creatine to range from 750 to 3.75 mCi mmol^−1^) and allowed to proceed for 6 min at 37°C. The uptake reaction was terminated after 6 min by washing the cell twice with 1 mL ice‐cold Krebs‐HEPES buffer. Non‐specific uptake was determined in presence of 300 μM GPA. Cells were lysed with 1% SDS (0.1 mL), the lysates were transferred to vials containing 2 mL of scintillation fluid, and radioactivity was determined by liquid scintillation counting. Uptake at the highest specific activity amounted to 3000–5000 counts per minute (cpm) and progressively declined to 100 cpm at the lowest specific activity.

### 5.8 Immunoblotting

Forty‐eight hours after transfection, HEK293 cells were washed twice with ice‐cold phosphate buffered saline (PBS) and subsequently lysed in a buffer containing 50 mM Tris·HCl, pH 8.0, 150 mM NaCl, 1% dodecylmaltoside, 1 mM ethylenediaminetetraacetic acid (EDTA), and protease inhibitors (Roche Complete™). The lysates were rotated at 4°C for 1 h. The insoluble material was removed by centrifugation at 13,000*g* for 30 min at 4°C. Aliquots of the lysate (protein content 30 μg) were mixed with reducing SDS‐loading buffer, heated for 30 min at 45°C and applied onto denaturing gel polyacrylamide gels (10% monomer concentration). After electrophoretic separation, the resolved proteins were transferred onto nitrocellulose membranes. The membranes were blocked with 5% skimmed milk in Tris‐buffered saline containing 0.1% Tween 20 (TBST) and then incubated with a rabbit polyclonal antibody directed against GFP (1:5000 dilution in TBST) and Gβ (1:500 dilution in TBST) overnight at 4°C. The immunoreactivity was detected by fluorescence detection using a donkey anti‐rabbit secondary antibody at 1:5000 dilution.

## AUTHOR CONTRIBUTIONS

**Thomas Stockner:** Conceptualization; methodology; project administration; writing – review and editing; funding acquisition; validation; supervision; resources. **Amy Clarke:** Software; validation; formal analysis; investigation; data curation; writing – original draft; writing – review and editing; visualization. **Daniel Szöllősi:** Software; writing – review and editing. **Sonja Sucic:** Methodology; resources; funding acquisition. **Ali El‐Kasaby:** Investigation. **Clemens V. Farr:** Investigation. **Michael Freissmuth:** Methodology; resources; data curation; writing – review and editing; supervision; funding acquisition.

## CONFLICT OF INTEREST STATEMENT

The authors declare no competing interests.

## Supporting information


**Data S1.** Supporting information.

## Data Availability

All raw data have been deposited at zenodo.org (https://doi.org/10.5281/zenodo.8154817).
