# Supplementary material for: Probing binding and occlusion of substrate in the human creatine transporter‐1 by computation and mutagenesis
Source: Protein Sci. 2024 Jan 1;33(1):e4842. doi: 10.1002/pro.4842 (PMC10751730; doi:10.1002/pro.4842)
Supplement: Supplementary file 1 — Data S1. Supporting information. [file PRO-33-e4842-s001.docx]

Probing binding and occlusion of substrate in the human creatine transporter-1 by computation and mutagenesis

*Running title:*

Probing substrate-triggered CRT1 occlusion

*Authors:*

Amy Clarke^1^, Clemens V. Farr^1^, Ali El-Kasaby^1^, Daniel Szöllősi^2^, Michael Freissmuth^1^, Sonja Sucic^1^, Thomas Stockner^1^*

*Affiliations:*

^1^ Institute of Pharmacology and the Gaston H. Glock Research Laboratories for Exploratory Drug Development, Center of Physiology and Pharmacology, Medical University of Vienna, Waehringerstr. 13A, 1090 Vienna, Austria

^2^ Department of Theoretical and Computational Biophysics, Max Planck Institute for Multidisciplinary Sciences, Am Fassberg 11, 37077 Göttingen, Germany

**Correspondance:*

Stockner Thomas.

Email: thomas.stockner@meduniwien.ac.at

*Address:* Institute of Pharmacology, Center of Physiology and Pharmacology, Medical University of Vienna, Waehringerstr. 13A, 1090 Vienna, Austria

*Tel.: +43 (0)1 40160-31215*

*Manuscript* without figures; 44 pages

- Figures 1-7 submitted as png images.

Supplementary Data 1: Multiple sequence alignment, docking results and table of Km values; 4 pages


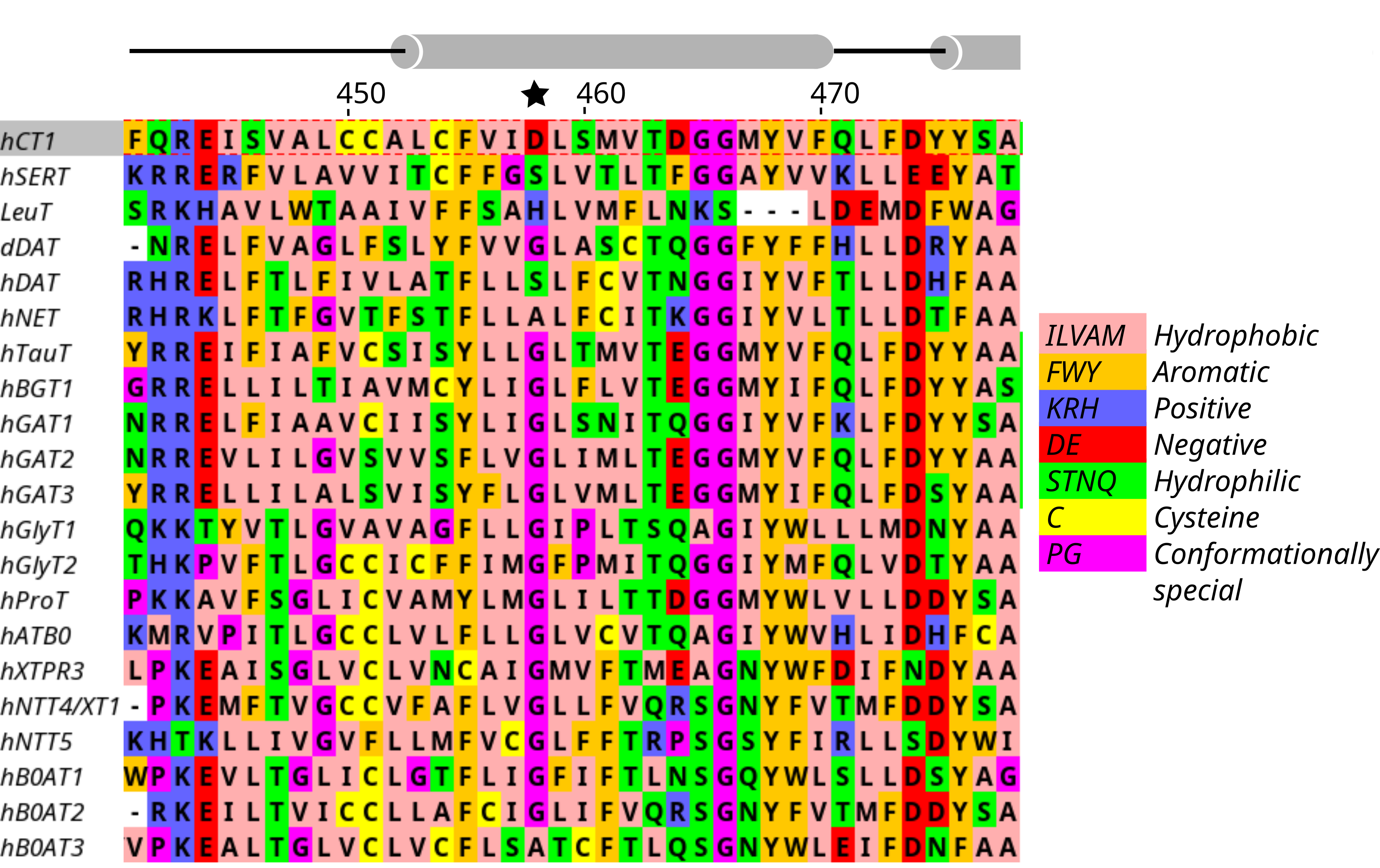


**Supplementary Figure 1: Multiple sequence alignment of TM9.** Multiple sequence alignment of TM9, with residue 458 indicated with a black star. The colors correspond to the zappo color scheme, where residues are colored according to their physicochemical properties.


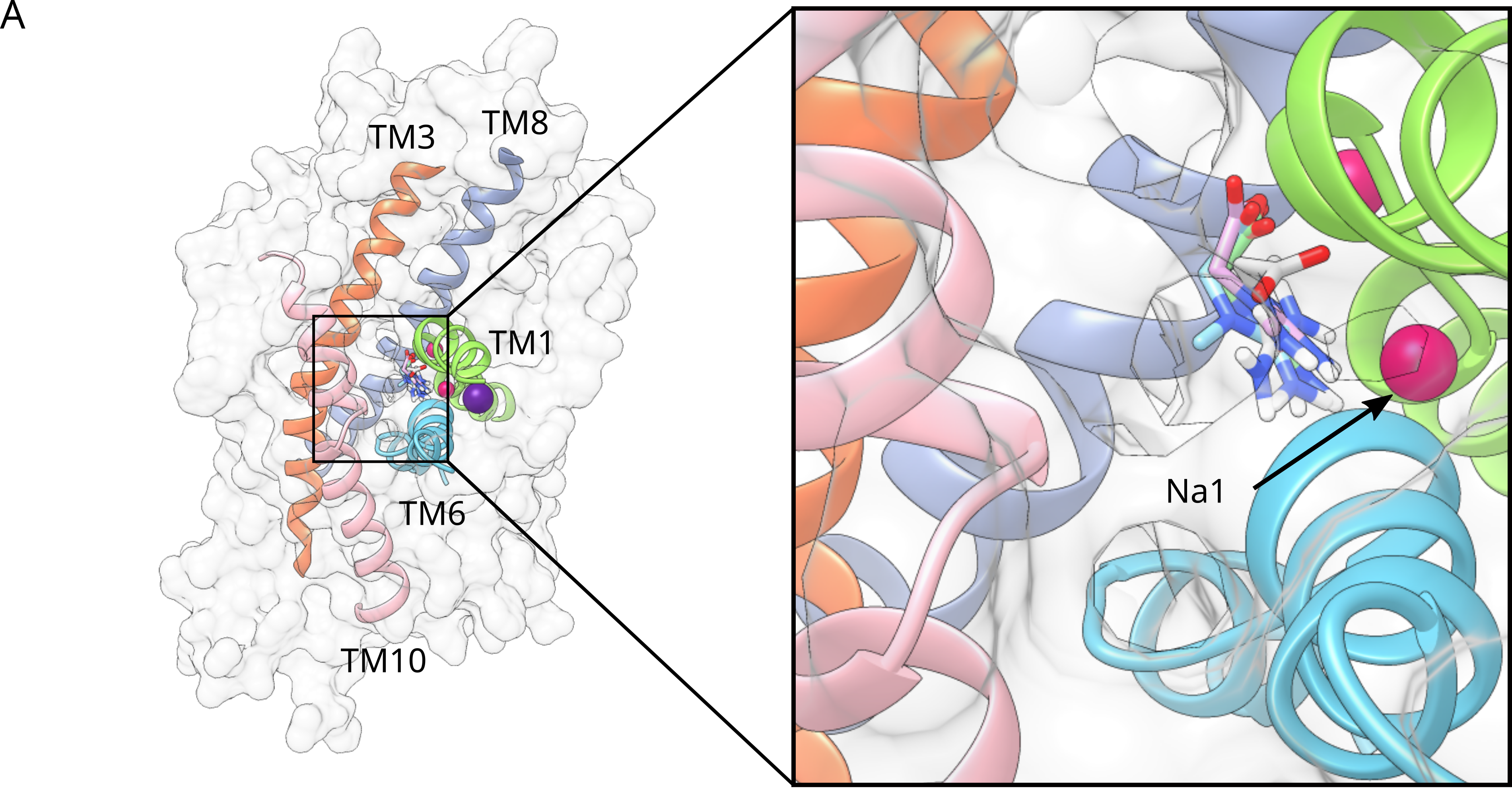
**Supplementary Figure 2: Docking with Autodock does not reproduce the cluster I bound pose. a)** The five highest scoring docked poses according to the Autodock scoring function. CRT1 is represented in a surface representation, with the helices lining the S1 also shown in a ribbon representation. The five creatine molecules are shown in a stick representation.

**Supplementary Table 1: Table of Km and Vmax values for WT-CRT1 and mutants.**

|  | Mean | Std Dev | SEM |
| --- | --- | --- | --- |
| **WT-CRT1** |  | | |
| Vmax (pmol/10^6^ cells/min) | 224.6 | 70.6 | 40.7 |
| Km (µM) | 19.4 | 2.4 | 1.4 |
| **CRT1-C144D** |  | | |
| Vmax  (pmol/10^6^ cells/min) | 148.7 | 22.4 | 12.9 |
| Km (µM) | 39.1 | 6.4 | 3.7 |
| **CRT1-C144S** |  | | |
| Vmax  (pmol/10^6^ cells/min) | 190.7 | 44.7 | 25.8 |
| Km (µM) | 29.3 | 6.9 | 4.0 |
